# A benchmarking study of two trauma centres highlighting limitations when standardising mortality for comorbidity

**DOI:** 10.1186/1749-7922-3-2

**Published:** 2008-01-16

**Authors:** Henry R Budd, Laurence M Almond, Peter A Oakley, Gilbert McKenzie, Peter Danne

**Affiliations:** 1 Trauma and Orthopaedics, Norfolk and Norwich University Hospital, Norwich, UK; 2 General and Colorectal Surgery, West Suffolk Hospital, Bury St. Edmonds, UK; 3 Intensive Care and anaesthesia, North Staffordshire Hospital, Stoke-on-Trent, UK; 4 Department of mathematics and statistics, Keele University, Staffordshire, UK; 5 Trauma and Orthopaedics, Royal Melbourne Hospital, Melbourne, Australia

## Abstract

**Introduction:**

A continuous process of trauma centre evaluation is essential to ensure the development and progression of trauma care at regional, national and international levels. Evaluation may be by comparison between pooled datasets or by direct benchmarking between centres. This study attempts to benchmark mortality at two trauma centres standardising this for multiple case-mix factors, which includes the prevalence of individual background pre-existing diseases within the study population.

**Methods:**

Trauma patients with an Injury Severity Score (ISS) >15 admitted to the two centres in 2001 and 2002 were included in the study with the exception of those who died in the emergency department. Patient characteristics were analysed in terms of 18 case-mix factors including Glasgow Coma Scale on arrival, Injury Severity Score and the presence or absence of 9 co-morbidity types, and patient outcome was compared based on in-hospital mortality before and after standardisation.

**Results:**

Crude mortality was greater at UHNS (18.2 vs 14.5%) with a non-significant odds ratio of 1.31 prior to adjusting for case-mix (P = 0.171). Adjustment for case mix using logistic regression analysis altered the odds ratio to 1.64, which was not significant (P = 0.069).

**Discussion:**

This study did not demonstrate any significant difference in the outcome of patients treated at either hospital during the study period. More importantly it has raised several important methodological issues pertinent to researchers undertaking registry based benchmarking studies. Data at the two registries was collected by personnel with differing backgrounds, in formats that were not completely compatible and was collected for patients that met different admissions criteria. The inclusion of a meaningful analysis of pre-existing disease was limited by the availability of robust data and sample size. We suggest greater communication between trauma research coordinators to ensure equivalent data collection and facilitate future benchmarking studies.

## Introduction

The outcome of trauma patients managed by individual centres is influenced by multiple factors including investment in the trauma service infrastructure, the facilities, technology and experience available at each centre, the type of injuries sustained and the background demographics of the patients managed. The performance of trauma centres has been evaluated by comparing outcomes with norms derived from pooled datasets, as in TRISS analysis and its modifications, and by benchmarking studies [1-3]. The logistic regression used in TRISS analysis uses few case-mix factors: age, Injury Severity Score, Glasgow Coma Score, systolic blood pressure and respiratory rate. Data collected by trauma centre registries are however generally more detailed than those submitted to pooled national databases and provide a richer set of case mix factors to allow a more discerning comparison.

A previous benchmarking study of trauma centre outcome compared the University Hospital of North Staffordshire (UHNS) with Oregon Health Sciences University (OHSU) in the United States [4]. The crude mortality analysis demonstrated a highly significant odds-ratio of 1.64 in favour of OHSU that was rendered non-significant after adjusting for case mix, which included twenty-three factors with equivalent definitions. The presence or absence of a significant past medical history was included as a binary variable (yes/no), providing this as the only indicator of co-morbidity with no qualification of severity or type. Adjusting case-mix for this binary indicator of co-morbidity does not accurately allow discrimination between the outcomes of two centres treating patients with a differing prevalence of significant pre-existing diseases, each of which can detrimentally effect the outcome of major trauma to a differing extent, as was demonstrated in a case control study by Morris et al [5]. In this study comparing two major trauma centres, one in the United Kingdom and one in Australia, we have attempted to account for the influence of pre-existing disease on mortality by including the prevalence of individual co-morbid factors within the case-mix adjustment.

### The University Hospital of North Staffordshire, Stoke-on-Trent, United Kingdom

The University Hospital of North Staffordshire (UHNS) is a large acute hospital with over 1000 beds. It has a local catchment population of 0.5 million and accepts tertiary referrals (except major burns) from a total of 1.7 million. The trauma research department collects data on all injured patients who stay longer than 72 hours in hospital, are admitted to the intensive care unit, die or are transferred from other hospitals. Co-morbidity data are routinely collected, but in non-mandatory fields.

### The Royal Melbourne Hospital, Melbourne, Australia

The Royal Melbourne Hospital (RMH) provides one of two adult major trauma services accepting tertiary referrals from the state of Victoria. It shares a total catchment population of over three million with the Alfred Hospital, Melbourne (AH). All surgical specialties (except for a burns service) are available on-site. Its trauma research department collects data for all patients who are admitted for longer than 24 hours or who die. Patient co-morbidity data are collected in a structured way, using the pre-defined conditions from the American College of Surgeons' trauma dataset [6].

## Methods

All patients admitted to the two centres between 1^st ^January 2001 and 31^st ^December 2002 with an Injury Severity Score (ISS) over 15 were identified, including those transferred from other hospitals. Patients who were dead on arrival or who died in the emergency department were excluded.

Data relating to eighteen case mix factors (excluding the nine co-morbidity factors) were extracted from both trauma registries after agreement that equivalent definitions were being used (table 1). Data for the co-morbidity factors were extracted directly from the trauma registry at RMH. At UHNS the original complete hospital clinical records were obtained and patients' co-morbidity was then classified retrospectively according to the forty six disease types routinely recorded by the RMH. A standardised form was used to record the presence or absence of each condition for every patient.

**Table 1:** Case Mix Factors

**Case-mix factors**
1. Date of admission
2. Age
3. Sex
4. Mechanism of injury
5. Type of trauma (*blunt/penetrating*)
6. Systolic blood pressure on arrival in primary hospital
7. GCS on arrival in primary hospital
8. Whether transferred from another hospital (*yes/no*)
9. Injury Severity Score

**Abbreviated Injury Scale (AIS) severity in the 9 body regions:**
10. Head AIS
11. Face AIS
12. Neck AIS
13. Chest AIS
14. Abdomen AIS
15. Spine AIS
16. Upper limbs AIS
17. Lower limb AIS
18. External region AIS

**Co-morbidity factors:**
19. Coronary artery disease (*yes/no*)
20. Hypertension (*yes/no*)
21. Non-insulin dependent diabetes mellitus (*yes/no*)
22. Psychiatric illness (*yes/no*)
23. Seizures (yes/no)
24. Stroke (yes/no)
25. Asthma (yes/no)
26. Drug abuse (yes/no)
27. Alcohol problem (yes/*no*)

### Statistical methods

A multiple linear logistic regression model, which allows the effects of continuous and categorical factors to be evaluated and weighted simultaneously, was used in a multi-factor analysis. Twenty-seven factors were studied, with automatic variable selection procedures (stepwise forward and backward algorithms) to suggest the best fitting logistic model. A 5% level of statistical significance was used to test statistical hypothesis and construct confidence intervals.

## Results

The RMH admitted 420 patients with an ISS >15 between 1^st ^January 2000 and 1^st ^January 2003, while during this period UHNS admitted 329 patients. Tables 2 to 4 show the frequency of individual factors.

**Table 2:** General Data

	Melbourne	Stoke
Number of cases	420	329
Direct admissions	222	252
Mean age	43.7 yr	40.5 yr
Sex	76% male	79% male
Blunt	92.9%	97.3%
Transfer	47.1%	23.4%
Mean number of co-morbidity factors	1.04	0.75
Mean systolic blood pressure	135.3	137.6
Mean GCS	10.5	10.8
Mean Injury Severity Score	24.2	27.3
Crude mortality	14.5%	18.2%

**Table 3:** Mechanism of Injury

	Melbourne	Stoke
Motor vehicle crash	59.8%	50.2%
Fall > 2 m	7.6%	17.3%
Fall < 2 m	18.6%	14.0%
Blows	6.9%	8.2%
Shooting	0.2%	1.2%
Stabbing	5.0%	0.9%
Other	1.9%	8.2%

**Table 4:** Co-morbidity Factors

	Melbourne	Stoke
Coronary artery disease	7%	6%
Hypertension	15%	9%
Non-insulin dependent diabetes	6%	2%
Psychiatric condition	9%	7%
Seizures	3%	5%
Stroke	3%	5%
Asthma	11%	12%
Drug abuse	15%	2%
Alcohol problem	11%	7%

Crude mortality was greater at UHNS (18.2 *versus *14.5%) yielding a crude odds ratio for death of 1.31 (P = 0.171). Adjustment for case mix altered the odds ratio to 1.64, but this was not statistically significant (P = 0.069) (table 5).

**Table 5:** Odds Ratios for Mortality Before and After Case Mix Adjustment

	Regression Coefficient	Standard Error	Wald	Degrees of Freedom	Significance	Odds Ratio
After	0.493	0.271	3.30	1	0.069	1.64
Before	0.272	0.199	1.87	1	0.171	1.31

## Discussion

This study is the first benchmarking trauma centre comparison to attempt to account for the prevalence of individual pre-existing diseases when calculating mortality rates of severely injured patients. Pre-existing disease in trauma patients is an independent risk factor for mortality, though some studies have indicated that it is less important than the mechanism of injury, the severity and site of injuries, and patient age [7-9]. The crude mortality rate was higher for Stoke than Melbourne (18.2% vs 14.5%) but while the odds ratio increased from 1.31 to 1.64 after standardisation for case-mix, including co-morbidity, this did not reach statistical significance.

The mean number of co-morbidity factors per patient was higher at RMH (1.04 versus 0.75), which may relate to differences in patient health behaviour, medical practice or simply by the completeness of co-morbidity recording in the two trauma registries. The incidence of the nine specific co-morbidities was similar with the exception of hypertension and chronic drug abuse, both of which were higher in the RMH population. A previous study looking at the presence of pre-existing conditions and mortality in older patients has shown hypertension to have a protective effect on outcome, possibly relating to the prescription of beta-blockers [7].

Less than 8% of major trauma (ISS>15) was penetrating in both centres, with RMH having double the incidence of UHNS. The lower rate of injuries in the 'external' region may reflect the presence of a nearby specialist burns unit in Melbourne, reducing the likelihood of primary transfer to RMH. Similarly, the slightly higher mean age at RMH may be due to the presence of a regional paediatric Major Trauma Service operated from Melbourne Royal Children's Hospital.

Victoria State (227,620 sq. km) is more than seventy-five times larger than the North West Midlands in the UK (2,997 sq. km). It would be surprising if the contrasting geographical features of the Australian catchment area did not have an influence on outcome. The proportion of major trauma patients transferred into RMH from primary hospitals was more than twice that transferred into UHNS, however in the analysis whether the patient was transferred or not was not a significant discriminator.

This study attempts to standardise mortality taking into account pre-existing disease but has several important limitations that merit discussion. The categorisation of co-morbidity was limited to a binary system relating to the presence or absence of individual co-morbidity and information regarding disease severity was not included due to the difficulty in quantifying this retrospectively. In addition, due to the size of the study populations only the 9 most prevalent disease types (of the 46 types recorded) could be included in analysis, excluding conditions such as cancer, renal failure and hepatic disease that have previously been shown to increase significantly the risk of death in elderly trauma patients [10]. Patient outcome is influenced by the nature and expediency of pre-hospital care, which we could not include within the case-mix standardisation because of the limited compatibility of available pre-hospital data recorded within the registries, and limited power due to population size. For inclusion of the above case-mix factors in a benchmarking study the sample size would have to be significantly larger if it were to prove a statistically significant difference in outcome. This would only be possible if individual trauma registries collected data for equivalent patients groups in a standardised format, and this was undertaken by staff with similar experience and training submitting information to the national registry. While this study has several methodological limitations it has highlighted the need for greater cooperation between trauma registry programme coordinators to ensure that data, including that relating to co-morbidity, is classified in a standardised format pertinent to future benchmarking studies necessary to drive global improvements in trauma care.

As trauma systems have improved, unexpected death rates have fallen making mortality a less discerning indicator of performance. Disability and morbidity are being heralded as better discriminators of outcome, although the validity of the current disability measures remains uncertain. As our ability to quantify outcome in this way improves, the importance of a robust analysis of co-morbidity becomes obvious.

## Competing interests

The author(s) declare that they have no competing interests.

## Authors' contributions

HB and LA participated in study design and data collection and coordinated the study. PA conceived the study and participated in study design. GM performed the statistical analysis. PD participated in data collection. All authors read and approved the manuscript.
